# Test Research on Seismic Performance and Shear Bearing Capacity of Assembled Composite Walls with Different Connections

**DOI:** 10.3390/ma19122549

**Published:** 2026-06-12

**Authors:** Xinwei Miao, Liyang Zhang, Liang Gu

**Affiliations:** 1 School of Science, Xi’an University of Architecture and Technology, Xi’an 710055, China; mxw2012@xauat.edu.cn; 2 State Key Laboratory of Green Building, Xi’an University of Architecture and Technology, Xi’an 710055, China; 3 Hebei Jianyan Technology Co., Ltd., Shijiazhuang 050227, China; basketballliang0@163.com

**Keywords:** welding connection, box connection, bolted connection, seismic performance, vertical connection, shear bearing capacity

## Abstract

To investigate the influence of dry connection methods on the seismic behavior of assembled composite walls, four assembled composite walls were designed and tested. Various dry connection techniques were adopted for the horizontal interfaces, namely sleeve grouting connection, welding connection, box connection, and bolted connection. The failure process, failure mode, bearing capacity, rigidity, steel bar strain, and energy absorption performance of the specimens were investigated through quasi-static cyclic loading tests. The results indicate that all types of connectors can effectively transfer loads and satisfy the conceptual design principle of “strong joint and weak component”. The damage evolution of the specimens is essentially identical, and the limiting drift angles all exceed 1/90. In addition, the shear resistance of the specimens with different connection methods is preliminarily analyzed and estimated.

## 1. Introduction

The connection technology of precast elements is a critical aspect of prefabricated shear wall structures. Through rational joint design, individual precast components are connected into an integrated system, achieving bearing capacity and durability comparable to those of cast-in-place concrete structures [1,2,3,4]. Currently, the connection methods for precast shear wall structures are mainly categorized into sleeve grouting connection, mortar anchor lap connection, dry connection, prestressed connection, and other types [5,6,7,8,9,10,11].

Dry connections are realized by embedding steel couplers or cast-in parts into prefabricated components and connecting them via bolts or welding. This approach offers the advantages of enhanced construction efficiency, reduced environmental impact, facilitated repair and post-earthquake replacement, and convenient installation in remote areas [12,13,14,15,16]. It represents a major research focus in assembly connection technology.

Vertical steel bar connection methods termed “box connection” and “bolted connection” have been explored. The results show that these reinforcement-to-wall-shoe connections exhibit substantial bearing capacity and deformation capacity. However, due to the yielding of the reinforcing bars and friction-grip bolts, residual joint opening occurs, producing a pronounced pinching effect under large lateral displacements [17,18,19,20,21,22]. The structural behavior of prefabricated shear walls equipped with bolted connections and rib beams of different heights has been studied. It is found that the failure of the wall is chiefly concentrated in the connection area. By optimizing the bolt performance and rib beam configuration, a pre-defined bolt failure mode in the connection area can be achieved, enabling post-earthquake replacement [23,24]. High-strength bolted steel frames are employed to join adjacent precast shear walls, forming a preassembled shear wall structure. The basic mechanical properties and load transfer mechanism of the lateral joint in this structure have been investigated. The results show that the deformability, plasticity, and energy dissipation capacity of the precast shear wall are marginally superior or equivalent to those of cast-in-place shear walls. It is also noted that the force transfer mechanism of the horizontal joint in the tension zone depends primarily on friction and dowel action, and the same mechanism governs the compression zone [23,25,26,27]. Multiple Slit Devices (MSD) are a type of energy dissipation connector used in prefabricated shear wall structures which dissipate energy through slotted steel plates. Seismic tests reveal that prefabricated shear walls incorporating this new connection device exhibit favorable energy dissipation and deformation capacity [28,29]. A horizontal ductile joint was designed using friction-grip bolts and low-yield-point (LYP) steel panels, and the deformability and energy absorption capacity of this structure are exceptional [30,31,32].

Two two-story assembled lateral load-resisting systems, connected by bolts and welding respectively, were designed and tested under quasi-static loading. The results show that both the bolted assembly and welded connection specimens exhibit good integrity and bearing capacity. In design, the detailing concept of “strong anchorage, weak steel plate” should be followed, and the fracture sequence of different components in the joint region should be controlled to ensure sufficient ductility and deformation capacity [5,13,33]. The welded connection using embedded steel plates is reliable; the fabricated columns demonstrate good seismic performance, and their ultimate bearing capacity is comparable to that of cast-in-place columns [34,35]. Nevertheless, the quality requirements for welded joints are stringent, and inadequate weld quality may lead to brittle failure [5,13]. Angle steel has also been used to weld the vertical reinforcement of the upper wall to the bottom plate, and this connection method can effectively transfer reinforcement stresses [36,37].

**Figure 1:**
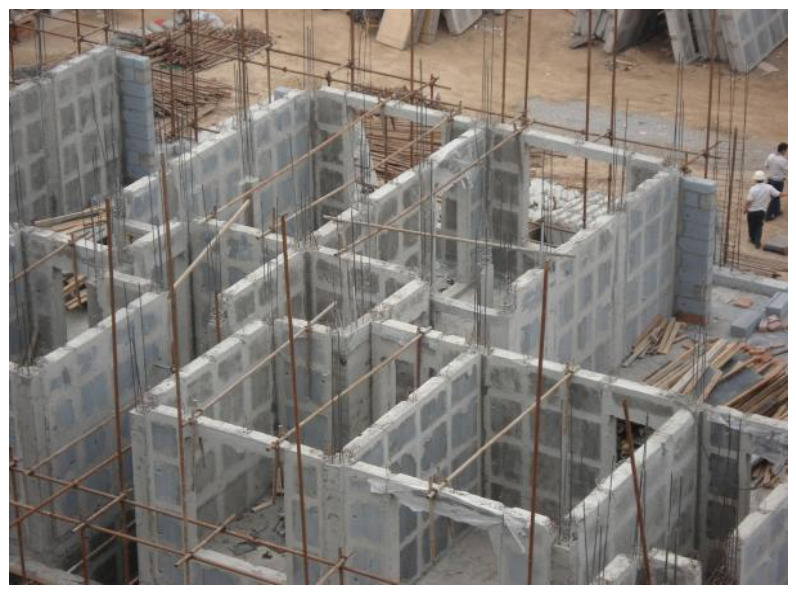
Assembled composite wall structure.

The fabricated composite wall system consists of fabricated composite walls, cast-in-place concrete restraining components, fabricated composite beams, laminated floor slabs, and other prefabricated components (Figure 1). Among these, the fabricated composite wall serves as the main lateral-force-resisting component, which is precast by filling cellular blocks within a concrete framework. The assembled composite wall grouted with sustainable materials can effectively reduce the consumption of raw materials. Moreover, owing to its material composition and unique structural configuration, the structural system possesses multiple seismic-resistant lines of defense [38,39,40].

Based on the above review of dry connections for prefabricated shear walls, three dry connection methods for the horizontal joints of assembled composite walls were proposed: welding connection, box connection, and bolted connection, targeting multi-story and low-rise assembled composite wall structures [41]. In addition, a wall panel with sleeve grouting connection was designed as a comparative specimen. The effect of different connection modes on the seismic response of the assembled composite wall was investigated, and a shear resistance formula for the specimens was proposed [42,43,44,45,46,47].

## 2. Experimental Program

### 2.1. Background Information

**Figure 2:**
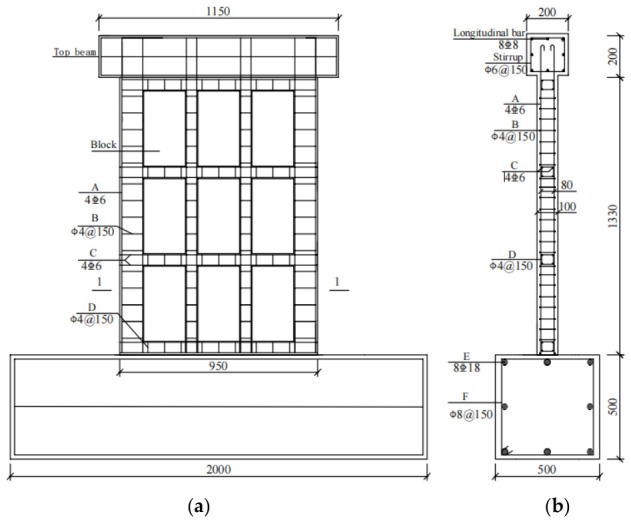

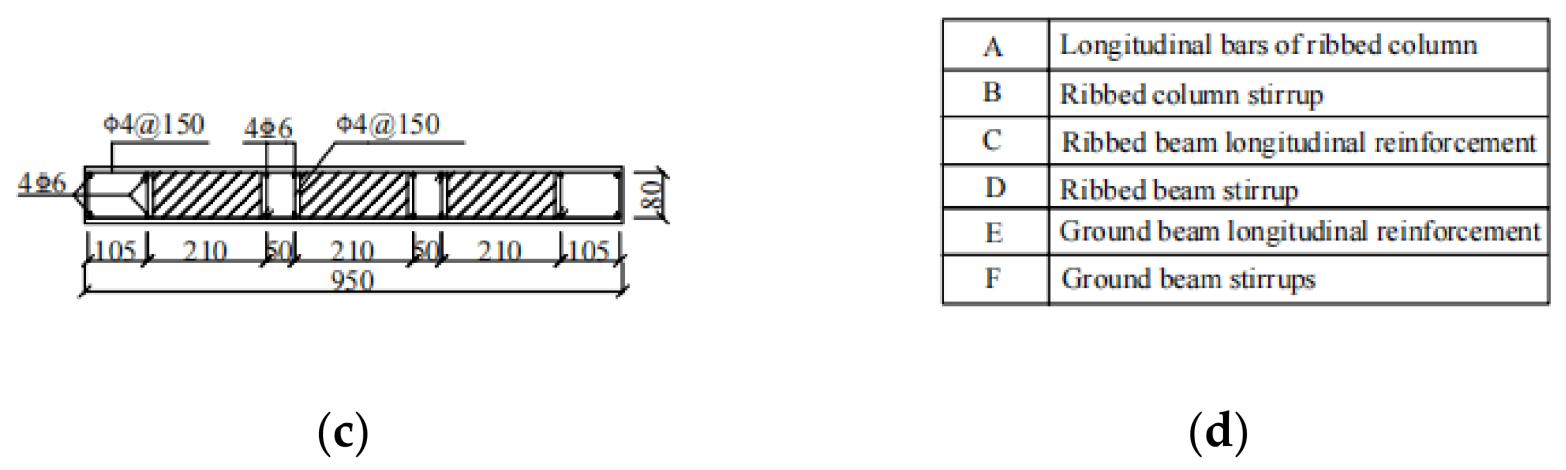
Cross-sectional dimensions and reinforcement of specimen. (**a**) Front elevation, (**b**) side elevation, and (**c**) 1-1 cross-section. (**d**) Table of codes and explanations for components are depicted in figure.

**Figure 3:**
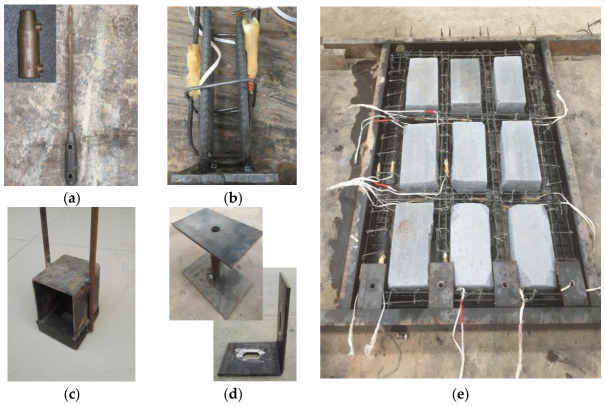
Detail of connection structure. (**a**) Sleeve grouting connection. (**b**) Welding connection. (**c**) Box connection. (**d**) Bolted connection. (**e**) Arrangement of connectors.

Four one-half-scale hybrid walls, each comprising a prefabricated bottom beam, a prefabricated wallboard, and a cast-in-place top beam, were designed. All specimens shared the same cross-sectional dimensions, reinforcement layout, and material properties. The wall size was 1340 (mm) × 950 (mm) × 100 (mm), and there was a 10 (mm) mortar layer at the wallboard bottom. The axial compression ratio was 0.2, and the dimensions and reinforcement details of the specimens are shown in Figure 2. The wall panels were connected to the bottom beam via four different dry connection methods: sleeve grouting, welding connection, box connection, and bolted connection, respectively. The connection details are illustrated in Figure 3, and the wall parameters are summarized in Table 1.

### 2.2. Material Characteristics

Six concrete cubes were cast during the pouring of the precast wallboards. The average recorded compressive strength of the concrete was 35.4 (MPa), and the measured compressive strength of the masonry blocks was 3.6 (MPa). All steel components were of Grade Q235, and the high-strength bolts were of Grade 8.8 s. HPB300 steel is used for stirrups, and HRB400 steel was used for longitudinal reinforcement. The measured yield strength ($f_{y}$) and ultimate strength ($f_{u}$) of the reinforcing bars and steel plates are listed in Table 2 [48].

### 2.3. Test Instrumentation and Testing Procedure

**Figure 4:**
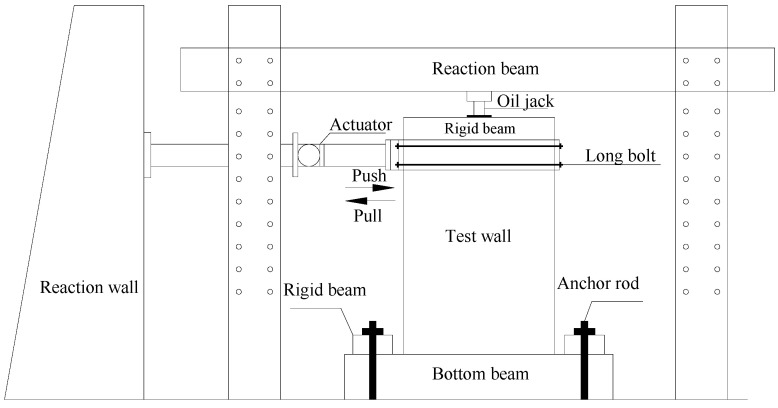
Test setup.

The vertical load was applied by a hydraulic jack mounted on the reaction beam and was uniformly distributed onto the prefabricated wallboard through a rigid distribution beam. The cyclic horizontal load was applied using a 1000 (kN) hydraulic actuator (Figure 4). A constant gravity load of 200 (kN) was determined based on an axial load ratio of 0.2. The axial load ratio is defined as N/($f_{c}A_{c}$). $A_{c}$ is the cross-section of all rib vertical members, $f_{c}$ is the crushing strength of concrete, N is the vertical load. In the testing procedure, the constant vertical load is first applied, and the reversed cyclic loads are then conducted on the specimens [49].

Prior to yielding, the cyclic loading was controlled by force, with each load step set at approximately 10 (kN) and cycled once. A force increment of 10 (kN), approximately 10% of the anticipated yield load, was adopted for each level, with one cycle per level. When a significant displacement developed and the bearing capacity dropped abruptly, the loading control was switched from force to displacement. Thereafter, each displacement increment was set at half of the displacement at the transition point, and each level was repeated for three cycles. The test was terminated when the lateral load dropped below 85% of the peak load.

### 2.4. Measurement Scheme

**Figure 5:**
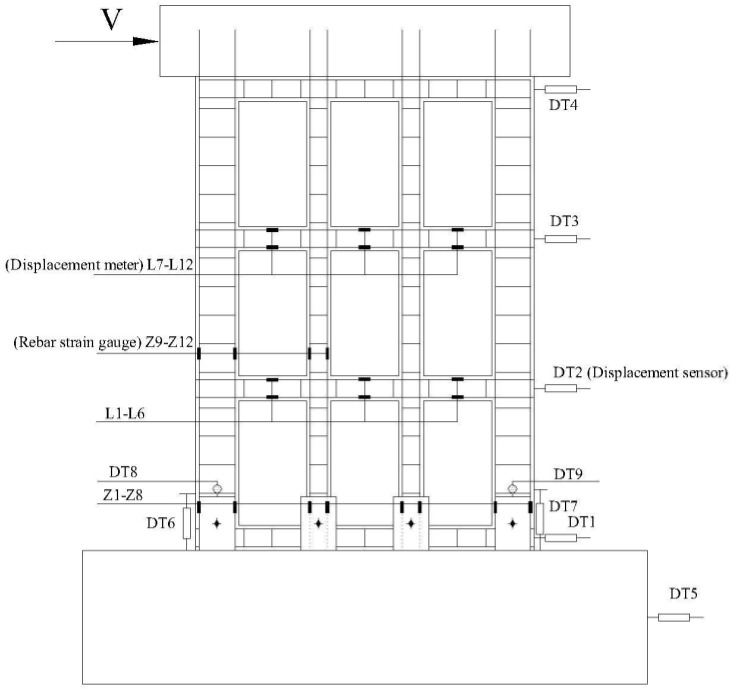
Arrangement of displacement transducers and strain gauges.

The following responses were measured during the test: the displacement at the top of the wallboard, the horizontal displacement at the height of the wall rib beams, the strain of the main reinforcement in the stiffener beams and vertical members, the horizontal displacement at the rib beam positions, the strain of the steel plates, and the relative slippage between the connectors and the wallboard in the bolted connection specimens. The instrumentation layout is shown in Figure 5. During the displacement-controlled loading stage, the sensors simultaneously recorded the horizontal load applied to the wall.

## 3. Failure Mode and Result Analysis

### 3.1. Failure Mode

**Figure 6:**
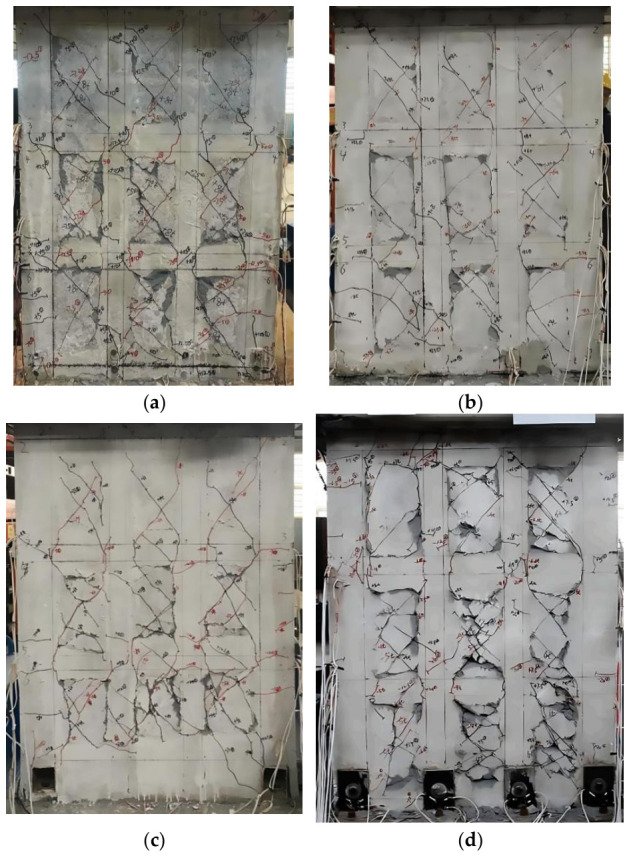
Failure modes of specimens. (**a**) GPCWH-1. (**b**) GPCWH-2. (**c**) GPCWH-3. (**d**) GPCWH-4.

**Figure 7:**
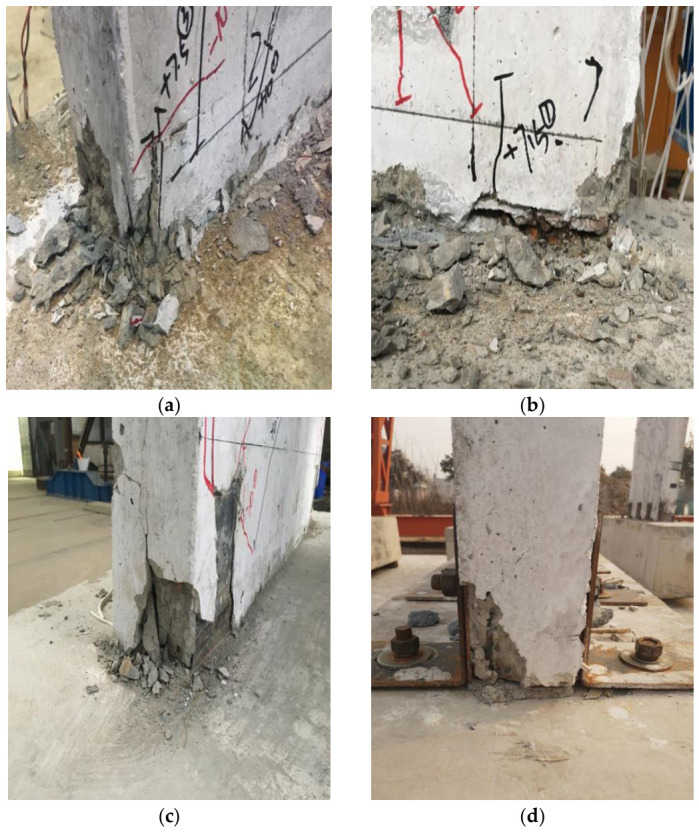
Damage of specimens. (**a**) GPCWH-1. (**b**) GPCWH-2. (**c**) GPCWH-3. (**d**) GPCWH-4.

The failure process and failure mode of all specimens were similar (Figure 6). The blocks in the compression zone cracked first, and the cracks gradually extended to the rib beams and middle rib columns, and finally to the side rib columns. At failure, the joint of GPCWH-2 was damaged due to insufficient weld quality (Figure 7b). The connectors of the other specimens remained intact, but the surrounding concrete was crushed. Taking GPCWH-3 as a representative example, the failure process is described as follows:

At a load of (θ=1/791), initial cracking appeared in the compression block at the lower part of the east side, marking the cracking point. With increasing horizontal load, the block cracks multiplied and extended. When the load was 60 (kN) (θ=1/443), the block cracks extended to the intermediate web beams. When loading to 80 kN (θ=1/296), all blocks were cracked, numerous cracks extended to the central web columns and rib beams, and a lateral crack appeared at the bottom of the west column. At this stage, the stiffness of the specimen decreased markedly, and load control was replaced by displacement control. At a lateral deflection of 7.5 (mm), the mortar bed at the bottom of the wallboard was damaged and the cracks in the side rib columns increased. At a lateral deflection of 10 (mm), cracks extended to the cast-in-place components, and a large number of blocks were broken and fell off. At a lateral deflection of 12.5 (mm), the concrete at the lower end of the rib columns was crushed (Figure 7c). When the lateral displacement reached (θ=1/66), the strength decreased to 85% of the peak load, and the test was terminated.

Overall, all specimens experienced three distinct stages: an initial elastic regime, a nonlinear deformation stage, and a collapse stage. In the pre-cracking stage, the specimens exhibited elastic behavior, and the first crack appeared within the blocks in the compressive region. With increasing load, the block cracks multiplied and extended to the stiffening beams and columns. In the failure stage, the interface between the embedded parts and the surrounding concrete cracked. Notably, no obvious damage or deformation occurred in the connectors, and no plastic deformation was observed in the bolts. The failure patterns of the joints are shown in Figure 7a–d.

### 3.2. Hysteretic Characteristics

**Figure 8:**
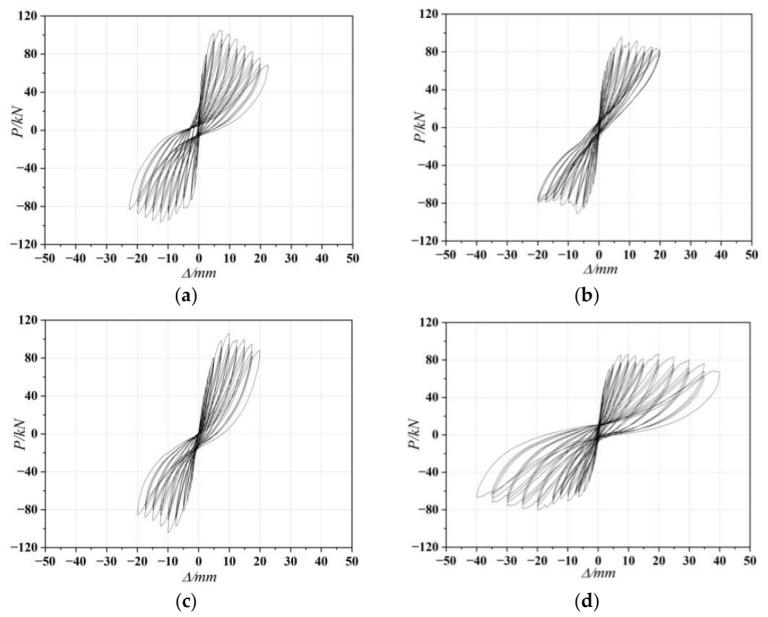
Hysteresis curve contrast of specimens. (**a**) GPCWH-1. (**b**) GPCWH-2. (**c**) GPCWH-3. (**d**) GPCWH-4.

The hysteretic curves of the specimens are shown in Figure 8. Overall, the hysteretic characteristics of all specimens were similar. Before yielding, the stiffness degradation was gradual, and the residual deformation upon unloading was minor. After yielding, the stiffness decreased rapidly with an increasing horizontal load, and the hysteresis loop area expanded, indicating enhanced energy dissipation.

For GPCWH-1 (SG-C), the first diagonal crack appeared in the bottom block on the tension side under a horizontal load of 60 (kN), corresponding to a cracking displacement of 1.12 (mm). As the load increased, the specimen gradually entered the elastic-plastic stage, and cracks propagated from the blocks to the rib beams and rib columns, accompanied by local concrete crushing and vertical cracking around the grouting holes. The test was terminated when the top lateral displacement reached 22 (mm). For GPCWH-2 (W-C), the rate of stiffness degradation accelerated after the peak load. At this point, owing to the weld failure, the horizontal shear force was primarily resisted by friction at the bottom of the wallboard, causing the wallboard to slide without sustaining further damage. Consequently, the area of the hysteretic response envelope was very small, and the energy dissipation capacity was the lowest among all specimens. The stiffness of GPCWH-3 (BX-C) decreased noticeably at the initial stage and the degradation accelerated after cracking. GPCWH-4 (BT-C) exhibited the largest hysteresis loop area; after reaching the peak load the bearing capacity declined gradually. Although extensive cracking occurred in the rib beams and columns, the wallboard retained sufficient load-carrying capacity, and its energy dissipation capacity was the highest. Due to the sliding of the wall panels, the ultimate displacement of GPCWH-4 (BT-C) was 1.5 to 1.8 times that of the other three specimens.

### 3.3. Skeleton Curve and Characteristic Point

**Figure 9:**
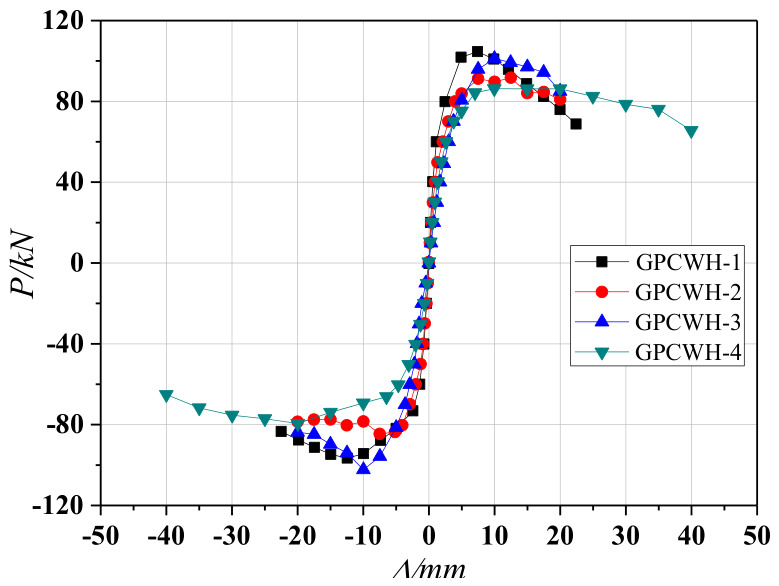
Skeleton curve contrast of specimens.

The skeleton curves of the test specimens are presented in Figure 9, and the corresponding deflections and loads at the characteristic points are summarized in (Table 3). The cracking point is defined as the moment when the first crack appears on the wall. The yield load is determined by the equivalent energy method, and the corresponding deflection represents the plastic displacement. On the skeleton curve, the point at which the load drops to 85% of the peak load is defined as the failure point. By averaging the characteristic points from the two loading directions, the following conclusions can be drawn:

(1) In the pre-cracking stage, the skeleton curves of all specimens are essentially identical, characterized by high stiffness and small residual deformation. After the peak load, the descending branches of the three dry-connected specimens are more gradual than that of GPCWH-1 (SG-C). For specimen GPCWH-2 (W-C), the load-carrying capacity dropped abruptly after the peak load because the poor weld quality caused a sudden failure in part of the weld, leading to a sudden drop in the load-carrying capacity.
(2) The order of specimens by bearing capacity, from highest to lowest, is: box connection, sleeve grouting connection, welding connection, and bolted connection. Compared with GPCWH-1 (SG-C), the yield load and peak load of GPCWH-2 (W-C) decreased by 7.2% and 7%, respectively; those of GPCWH-3 (BX-C) increased by 5.3% and 5.0%, respectively; and those of GPCWH-4 (BT-C) decreased by 13% and 17%, respectively.
(3) In terms of the displacement ductility coefficient, GPCWH-2 (W-C), GPCWH-3 (BX-C), and GPCWH-4 (BT-C) achieved 1.05 times, 0.66 times, and 1.8 times that of GPCWH-1 (SG-C), respectively. The excessively large deflection of the bolted specimen is attributed to the horizontal sliding of the wallboard.

### 3.4. Reinforcement Strain

**Figure 10:**
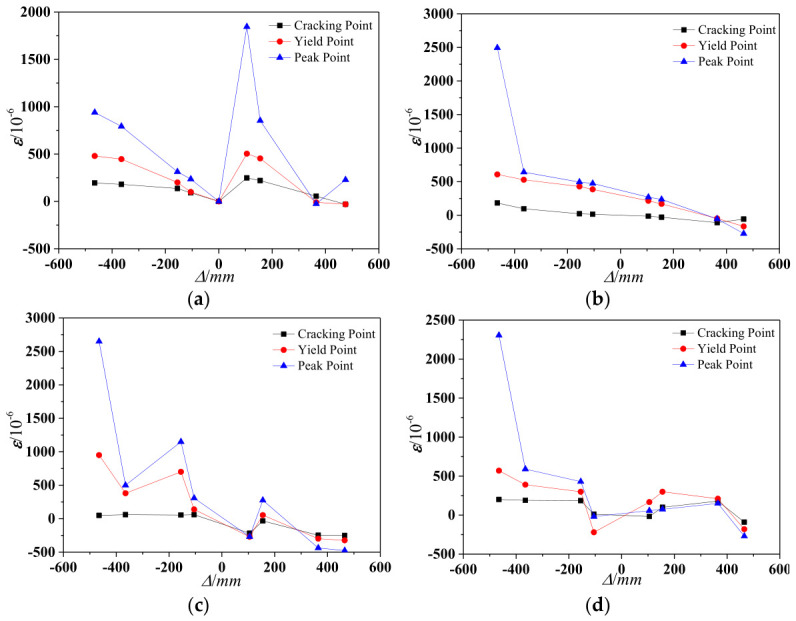
Strain analysis of rib reinforcements. (**a**) GPCWH-1. (**b**) GPCWH-2. (**c**) GPCWH-3. (**d**) GPCWH-4.

The strain distributions of the main reinforcing bars at different positions are shown in Figure 10. At the cracking point, the strain in the main reinforcement exhibited a nearly linear distribution along the cross-section, indicating that the elongation of the reinforcement generally conformed to the plane-section assumption before cracking. At the yield point, the increment of the longitudinal reinforcement strain was small, and specimen GPCWH-2 still satisfied the plane-section assumption. At the peak load, the reinforcement in the outermost rib columns yielded in all specimens except GPCWH-1.

### 3.5. Stiffness Degradation

**Figure 11:**
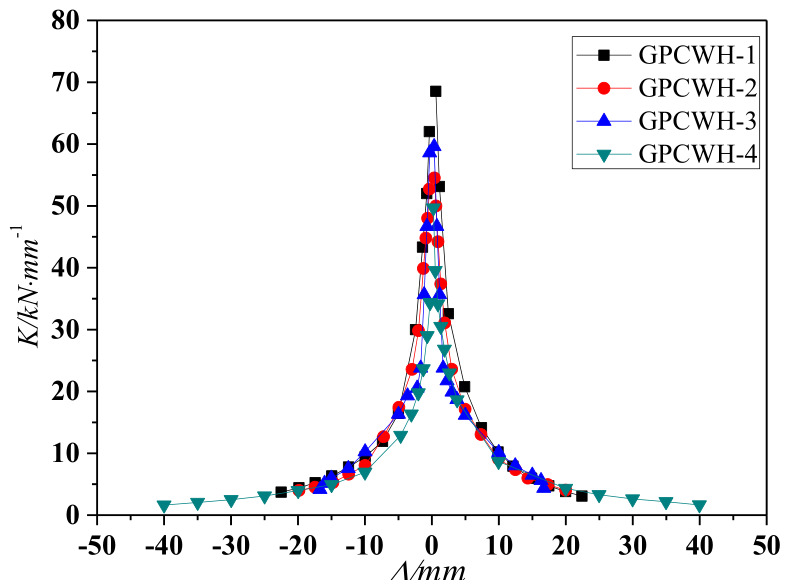
Secant stiffness degradation curves of specimens.

The secant stiffness of the wallboard was calculated according to reference [50], and the stiffness degradation curves are plotted in Figure 11. The initial stiffness of GPCWH-1 was the highest, being 1.15 times that of GPCWH-3, 1.22 times that of GPCWH-2, and 1.56 times that of GPCWH-4. The stiffness decreased with increasing lateral displacement, and the stiffness degradation curves of all specimens largely coincided at the later stage of loading.

### 3.6. Energy Dissipation Capacity

**Figure 12:**
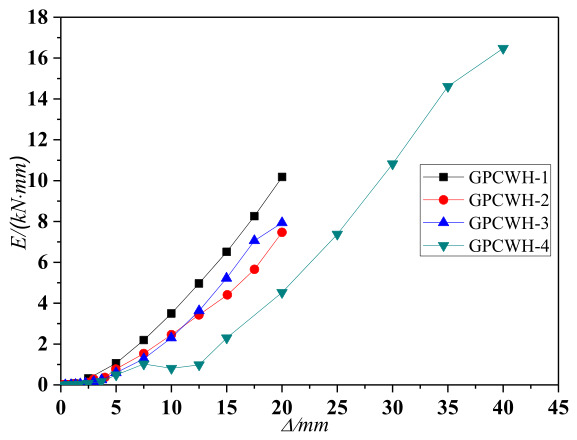
Energy dissipation curves of specimens.

The energy dissipation capacity of the specimens is primarily evaluated through the area enclosed by the hysteretic loops. The larger the enclosed area, the greater the energy dissipation capacity. In this study, the cumulative energy dissipation E is adopted to quantify the energy dissipation capacity of the test specimens. The relationship between the cumulative energy dissipation over three cycles and the horizontal displacement is shown in Figure 12.

With increasing lateral displacement, the cumulative energy dissipation of each specimen increased exponentially. At the same displacement level, the cumulative energy dissipation of GPCWH-1 was the highest, while that of GPCWH-2 was the lowest; the values of GPCWH-2 and GPCWH-3 were close to each other. However, owing to its superior ductility, the ultimate cumulative energy dissipation of GPCWH-4 was the highest, reaching approximately 162% of that of GPCWH-1.

## 4. Prediction of Shear Bearing Capacity

To study the connection form more deeply in the future, the diagonal tension strength of the assembled composite wall with various vertical connections should be preliminarily analyzed and calculated.

### 4.1. GPCWH-1 and GPCWH-3

As shown in Table 3, the load-carrying capacity of specimen GPCWH-3 (BX-C) is similar to that of specimen GPCWH-1 (SG-C). This similarity arises because both connection methods share the same fundamental principle: connecting the embedded bolts or the reinforcement of the lower component to the upper component through mechanical fastening or grouting. In the code [51], only the shear strength of the joint is considered for prefabricated shear walls with box connections. Given these considerations, the shear strength formula for box-connected walls is taken to be identical to that of sleeve grouting connections [52]. The formula is shown as Formula (1), where $f_{c}$ is the compression strength of the concrete, $A_{c}$ is the cross-sectional areas of the rib columns, $f_{q}$ refers to the design compressive strength of the blocks, $A_{q}$ refers to the sum of the cross-sectional areas of the blocks, $f_{y}$ is the tensile strength of the longitudinal reinforcement in the rib column, and $A_{s}$ is the cross-sectional area of the longitudinal reinforcement in the rib column.

(1) $$V=\frac{1}{\left(\lambda -0.5\right)}\left[(0.03f_{c}A_{c}+0.03f_{q}A_{q}+0.1N)+f_{y}A_{s}\right]$$

### 4.2. GPCWH-2 and GPCWH-4

The bearing capacity and stiffness of these two connection types for the assembled composite walls are notably reduced. This suggests that the bolts reduce the shear strength of the wallboard, or that the wallboard does not reach its full bearing capacity [53].

In reference [53], it is proposed that in the post-yield stage, the load-resisting mechanism of the wall can be idealized as a strut-and-tie model, which serves as the basis for estimating the ultimate shear capacity of the wall, following the principle of superposition. In the formula (as shown in Formula (1)) there is a separate item to consider the effect of the welding plate on the shear bearing strength. According to the mechanism of dowel action across the shear interface, when the bottom of the wallboard is subject to shear force, the dowel shear transfer for setting the steel bar of the welded plate rib column is $V_{d}=0.43\;A_{\mathrm{src}}f_{\mathrm{yrc}}/\sqrt{3}$, α=0.43 is the influence coefficient of the welding connection, *f_yrc_* is the design strength of the longitudinal reinforcement of the rib column; $A_{src}$ is the cross-sectional area of the longitudinal reinforcement of the rib column. $f_{crc}$ is the compressive strength of the rib column concrete, $A_{crc}$ refers to the cross-sectional area of the rib column concrete, $f_{yf}$ is the tensile strength of the reinforcement in the outer frame column, and $A_{sf}$ refers to the cross-sectional area of the reinforcement in the outer frame column.

(2) $$V=\frac{1}{\left(\lambda -0.5\right)}\left[0.09f_{b}A_{b}+0.1f_{c}A_{c}+0.18f_{crc}A_{crc}+0.02N\right]+0.14f_{yf}A_{sf}+0.11(n-2)f_{yrb}A_{srb}+0.43f_{yrc}A_{src}/\sqrt{3}$$

For specimen GPCWH-2, Formula (2) can be used to calculate the shear capacity of the specimen, $0.18f_{\mathrm{crc}}A_{\mathrm{crc}}$ and $0.14f_{\mathrm{yf}}A_{\mathrm{sf}}$ are the influence items of the concrete and reinforcement of cast-in-place structural columns, *f_b_* is the compression strength of the block, $A_{b}$ and $A_{c}$ are the cross-sectional areas of rib columns and blocks, $f_{yrb}$ and $A_{srb}$ are the tensile capacity and area of the longitudinal rebar of the rib beams, *n* is the number of rib beams, and λ is the shear span ratio of the test piece. The shear strength of specimen GPCWH-2 adopts Formula (3).

(3) $$V=\frac{1}{\left(\lambda -0.5\right)}\left[0.09f_{b}A_{b}+0.1f_{c}A_{c}+0.02N\right]+0.11(n-2)f_{yrb}A_{srb}+0.43f_{yrc}A_{src}/\sqrt{3}$$

In reference [54], through establishing FEM under the monotonic loading test, the effect of various variables on the load-carrying capacity of the wallboard is studied. Moreover, the equation of the shear load-carrying capacity of the wallboard was proposed. The effect of the fastener on the shear load-carrying capacity of the wall is $V=0.03Nf_{cpy}A_{cpy}$, *n* is the number of the connectors, and $f_{cpy}$ and $A_{cpy}$ are the yield strength and section area of the steel plate. The shear strength of specimen GPCWH-4 adopts Formula (4).

(4) $$V=\frac{1}{\left(\lambda -0.5\right)}[0.09f_{b}A_{b}+0.1f_{c}A_{c}+0.02N]+0.11(n-2)f_{yrb}A_{srb}+0.03nf_{cpy}A_{cpy}$$

The shear capacity of each test piece is determined by (Table 4). The results illustrate that the formula value is comparable to the test value, and the error between the two results is less than 10%.

### 4.3. Conditions

When $N>0.2f_{c}A_{c}$, $N=0.2f_{c}A_{c}$. When $N\leq 0.2f_{c}A_{c}$, the actual calculated value should be used. N is the axial force.

When λ<1.5, λ=1.5, when λ>2.5, λ=2.5, and when 1.5<λ<2.5, the real determined value should be used. λ is the Aspect ratio [55].

## 5. Conclusions

(1) In the test, the connectors of all specimens remained undamaged, indicating that the dry connection methods are reliable in design and provide good seismic performance. Furthermore, all connections satisfy the seismic design principle of “strong joint, weak component”.
(2) The welded connection specimen exhibited rapid stiffness degradation, limited plastic deformation, and low energy dissipation capacity. Moreover, if the weld quality is inadequate, brittle failure of the joint is likely to occur. The box connection offers high bearing capacity and gradual stiffness degradation, but its deformation capacity is limited. The bolted connection exhibits the lowest bearing capacity but the highest deformation capacity and the best energy dissipation performance.
(3) The structural capacity, deformability, and energy dissipation capacity of the test specimens are significantly influenced by the type of vertical connection. Compared with the sleeve-grouted joint specimen (GPCWH-1), the welded connection specimen (GPCWH-2) showed a 5% increase in displacement ductility coefficient, a 7% decrease in bearing capacity, and a 22% decrease in cumulative energy dissipation; the box connection specimen (GPCWH-3) showed a 33% decrease in displacement ductility coefficient, a 5% increase in bearing capacity, and a 26.7% decrease in cumulative energy dissipation; the bolted connection specimen (GPCWH-4) showed an 80% increase in the displacement of the ductility coefficient, a 17% decrease in bearing capacity, and a 62% increase in cumulative energy dissipation.
(4) The proposed formulas for the bearing capacity of the specimens can serve as a reference for preliminary design and estimation.
(5) It should be noted that the conclusions drawn in this study are based on a limited number of specimens and specific loading conditions. The effects of long-term cyclic loading, construction tolerances, material deterioration, and varying geometric parameters were not considered. Therefore, further experimental and numerical investigations are required to validate the applicability of the proposed connection methods and design formulas under more comprehensive conditions.

## Figures and Tables

**Table 1:** Parameter of specimens.

No.	Connection Mode
GPCWH-1	Sleeve grouting connection (SG-C)
GPCWH-2	Welding connection (W-C)
GPCWH-3	Box connection (BX-C)
GPCWH-4	Bolted connection (BT-C)

**Table 2:** Mechanical properties.

	Yield Strength (MPa)	Tensile Strength (MPa)
HPB300	367	406
HRB400	437	555
Q235	335	455

**Table 3:** Load and placement of specimens at characteristic points.

No.	Direction	Cracking Point			Yield Point			Peak Point			Failure Point			$\Delta_{u}/\Delta_{y}$
		$P_{\mathrm{cr}}/\mathrm{kN}$	$\Delta_{\mathrm{cr}}/\mathrm{mm}$	$\theta_{\mathrm{cr}}$	$P_{y}/\mathrm{kN}$	$\Delta_{y}/\mathrm{mm}$	$\theta_{y}$	$P_{p}/\mathrm{kN}$	$\Delta_{p}/\mathrm{mm}$	$\theta_{p}$	$P_{u}/\mathrm{kN}$	$\Delta_{u}/\mathrm{mm}$	$\theta_{u}$	***μ***
GPCWH-1	push	60	1.1	1/1187	88.1	3.1	1/433	104.6	7.4	1/179	88.7	14.9	1/89	4.85
	pull	60	1.3	1/964	81.0	4.4	1/302	96.5	12.4	1/107	81.9	22.04	1/60	5.02
GPCWH-2	push	50	1.3	1/1000	79.9	4.0	1/221	95.6	7.3	1/181	81.4	19.98	1/67	4.98
	pull	50	1.2	1/1064	77.6	3.7	1/357	92.2	7.2	1/185	77.5	19.91	1/67	5.35
GPCWH-3	push	50	1.6	1/791	88.4	5.8	1/226	102.6	9.8	1/135	84.9	19.98	1/67	3.40
	pull	50	2.2	1/601	90.0	6.2	1/213	103.3	9.8	1/134	83.7	20.1	1/66	3.21
GPCWH-4	push	40	2.6	1/509	71.7	4.3	1/307	86.2	9.95	1/134	65.6	39.96	1/33	9.24
	pull	40	2.0	1/655	73.9	4.6	1/285	80.6	19.78	1/67	65.7	40.1	1/33	8.58

**Table 4:** Comparison of theoretical and experimental values.

**No.**	**Direction**	**Formula/kN**	**Test/kN**	**Error/** **%**
GPCWH-1	Push	101.1	104.6	−3.4
	Pull	101.1	96.5	4.8
GPCWH-2	Push	101.3	95.6	6.0
	Pull	101.3	92.2	9.7
GPCWH-3	Push	101.1	102.6	−1.3
	Pull	101.1	103.3	−2.2
GPCWH-4	Push	88.6	86.2	3.9
	Pull	88.6	80.6	9.8

## Data Availability

The original contributions presented in this study are included in the article. Further inquiries can be directed to the corresponding author.
